# Cardiovascular Safety in Postmenopausal Women and Men With Osteoporosis Treated With Denosumab and Zoledronic Acid: A Post‐Authorization Safety Study

**DOI:** 10.1002/jbm4.10793

**Published:** 2023-08-21

**Authors:** Leslie Spangler, Carrie M. Nielson, M. Alan Brookhart, Rohini K. Hernandez, Robert Kees Stad, Tzu‐Chieh Lin

**Affiliations:** ^1^ Center for Observational Research, Amgen Inc. Thousand Oaks CA USA; ^2^ NoviSci, Inc. Durham NC USA; ^3^ Department of Population Health Sciences Duke University Durham NC USA; ^4^ Research and Development, Amgen Inc. Europe GmbH Rotkreuz Switzerland

**Keywords:** ANTIRESORPTIVES, OSTEOPOROSIS, THERAPEUTICS—OTHER

## Abstract

Osteoporosis and cardiovascular disease are common in older adults. Treatment of osteoporosis reduces the burden of debilitating fractures; however, it is important to understand the benefit versus risk of treatment. This study evaluates the risk of stroke (ischemic or hemorrhagic) and myocardial infarction (MI) among postmenopausal women and men initiating osteoporosis treatment with denosumab (receptor activator of nuclear factor κB ligand [RANKL] inhibitor) or zoledronic acid (bisphosphonate) between October 2010 and June 2019. A retrospective cohort study employing the new user/active comparator design was conducted. Analyses were conducted separately in two national US commercial databases, MarketScan® and Optum® for reproducibility. Inverse probability of treatment and censoring weighting was employed to control for confounding and informative censoring. Cumulative risks at 6‐month, 12‐month, and 36‐month time points were calculated and adjusted risk ratios and differences (with 95% confidence intervals [CIs]) were estimated. In MarketScan® and Optum® databases, 96,611 and 73,127 patients met all study eligibility criteria, respectively. At 36 months, the risk ratio estimates (zoledronic acid referent group) were 1.22 (95% CI, 0.77–1.66) and 0.97 (95% CI, 0.63–1.32) for MI and 1.00 (95% CI, 0.61–1.40) and 0.87 (95% CI, 0.56–1.17) for stroke in MarketScan and Optum, respectively. Most of the treatment associations across the other time periods and outcomes also had 95% CIs including the null value. In these large samples of real‐world US patients, no increased risk in MI and stroke were identified for up to 36 months of treatment in denosumab users compared with zoledronic acid users. © 2023 Amgen. *JBMR Plus* published by Wiley Periodicals LLC on behalf of American Society for Bone and Mineral Research.

## Graphical abstract

This study evaluates the risk of stroke (ischemic or hemorrhagic) and myocardial infarction (MI) among postmenopausal women and men initiating osteoporosis treatment with denosumab (RANKL inhibitor) or zoledronic acid (bisphosphonate). A retrospective cohort study employing the new user/active comparator design was conducted in two national US commercial databases. No increased risk in MI and stroke were identified for up to 36 months of treatment in denosumab users compared with zoledronic acid users.

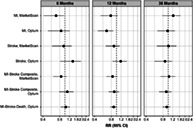

## Introduction

Osteoporosis is a common condition of aging that can lead to debilitating fractures and attendant economic and societal burden.^[^ ^1^ ^]^ Osteoporosis patient populations are primarily postmenopausal women and—in much smaller numbers—men older than 50 years. In these populations, the risk of cardiovascular and cerebrovascular events is a significant concern. Drug therapies based on bone biology can reduce fracture risk. However, a careful evaluation of the cardiovascular benefit versus risk is warranted, especially with respect to drugs that can affect calcium metabolism, such as osteoporosis treatment, because vascular calcifications is related to an increased risk of cardiovascular disease.^[^ ^2^, ^3^ ^]^

Denosumab is a fully human monoclonal antibody against receptor activator of nuclear factor κB ligand (RANKL). It has been proposed that vascular calcification relies on the osteoprotegerin (OPG)/RANKL signaling pathway; however, the exact mechanism remains speculative.^[^ ^4^ ^]^ Although bisphosphonates have been evaluated for potential atherosclerotic protection as well as for increased risk of atrial fibrillation, findings are inconclusive.^[^ ^3^, ^5^, ^6^, ^7^, ^8^, ^9^, ^10^ ^]^ In trials comparing denosumab with bisphosphonate therapy, small numerical imbalances in investigator‐reported cardiovascular and cerebrovascular events were reported.^[^ ^11^, ^12^ ^]^ Hence, European Union Regulators requested an evaluation of the potential risk of cardiovascular and cerebrovascular events in a real‐world population of denosumab users treated for osteoporosis. In this study, we evaluated the risk of cardiovascular (myocardial infarction [MI]) and cerebrovascular (ischemic or hemorrhagic stroke) events among postmenopausal women and men with osteoporosis initiating treatment with two different classes of antiresorptives: denosumab (a RANKL inhibitor) or zoledronic acid (a bisphosphonate).

## Patients and Methods

### Study design

A retrospective cohort study was conducted to evaluate the risk of cardiovascular and cerebrovascular events among postmenopausal women and men initiating osteoporosis treatment with denosumab or zoledronic acid. An active comparator, new user, as‐treated design was used, with the index event defined as initiation of either therapy.^[^ ^13^, ^14^ ^]^ Confounding by covariates at initial treatment choice was controlled using propensity score modeling and inverse probability of treatment weighting (IPTW).^[^ ^15^, ^16^ ^]^ Similarly, potentially informative censoring was addressed using censoring weighted estimation functions.^[^ ^17^ ^]^

The protocol was submitted to the European Medicines Agency (EMA) before the conduct of the study and the protocol is posted on ENCePP (https://www.encepp.eu/encepp/viewResource.htm?id=42980). To further ensure the integrity of the study, we followed a staged approach to assessing covariate balance before proceeding to outcome evaluation.^[^ ^18^ ^]^ Investigators who provided input during the study on propensity score model specifications and adequacy of covariate balance were prevented from accessing outcome data to avoid bias from knowledge about how analytic decisions might affect treatment effect estimates.

### Data sources

Analyses were conducted separately in two national US commercial databases, International Business Machines (IBM) Watson Health (formerly Truven) MarketScan Commercial including Medicare Supplemental and Coordination of Benefits Databases and Optum Clinformatics® Data Mart. Both claims databases represent the medical experience of insured employees and their dependents and patients with Medicare supplemental insurances. They include information on eligibility, pharmacy, procedure, and medical claims data (inpatient and outpatient). The MarketScan database covers approximately 17 million lives annually, covered under a variety of fee‐for‐service and capitated health plans. The Optum data are spread across all 50 US states and are racially/ethnically diverse. Optum data include death dates from several sources, including the Social Security Administration (SSA) Death Master File. All analyses were based on these deidentified secondary data sources. No primary data collection occurred; patient consent and institutional review board approval were not needed.

### Study population

Patients were included in the analyses if they were aged ≥55 years at the index date, defined as the first date of administration of study drug between October 1, 2010, and June 30, 2019, and had at least 455 days of continuous enrollment in the data source preceding the index date (ie, baseline period). Because zoledronic acid is given yearly, a minimum 455‐day (ie, approximately 15‐month) baseline period enabled assessment of its past use.^[^ ^19^ ^]^ Patients were excluded from the study if they were diagnosed with Paget's disease or cancer (or were receiving treatment for cancer) or had a history of stroke or MI events during the baseline period. Patients were excluded for previous administration of study medications (denosumab or zoledronic acid) before the index date. Look‐back for previous study medication included all available data.^[^ ^20^ ^]^

### Treatment groups

The standard dosing interval for denosumab 60 mg is once every 6 months (ie, 182 days). The osteoporosis standard dosing interval for zoledronic acid is 5 mg once a year (ie, 365 days). Patients were considered continuously exposed to treatment from the date of first administration through the end of an allowable gap period of 60 days after the standard dosing interval following the last recorded administration. Codes used to identify treatments are included in the Supporting Information.

### Endpoints

Cardiovascular endpoints were defined using validated algorithms that have also been used in previous studies of MI and stroke.^[^ ^21^, ^22^, ^23^, ^24^ ^]^ MI was identified with a hospital discharge diagnosis code of acute MI using International Classification of Diseases Ninth Revision (ICD‐9) codes (410.x0, 410.x1) and ICD 10th Revision (ICD‐10) codes (I21, I21.0, I21.0x, I21.1, I21.1x, I21.2, I21.2x, I21.3, I21.4, I21.9). Stroke was defined with a hospital discharge diagnosis code of ischemic or hemorrhagic stroke using ICD‐9 codes (430, 431, 433.x1, 434.x1, 436) and ICD‐10 codes (I60.xx, I61.xx, I63.0xx‐I63.6xx, I63.8, I63.9). We also assessed (i) the composite endpoint of MI and stroke in each database and (ii) the composite endpoint of MI, stroke, and all‐cause mortality in the Optum database, which included complete information on death (MarketScan data include information on in‐hospital deaths only). The primary time point for estimation of cumulative risks, risk ratios (RRs), and risk differences (RDs) was 36 months; however, 6‐month and 12‐month time points were also evaluated. To estimate the per protocol effect, patients were censored by treatment discontinuation or treatment change. Patients were also censored by loss of health plan enrollment, end of available data, and in the Optum cohort by death.

### Covariates

Selected covariates included demographics, risk factors (including diseases and medications) for cardiovascular/cerebrovascular outcomes and/or osteoporosis, healthcare utilization, and year of cohort entry were used for the description for patients starting each therapy and for propensity score analysis. Covariates were assessed during the baseline period. They were identified using algorithms of inpatient and outpatient ICD‐9 Clinical Modification (ICD‐9 CM)/ICD‐10‐CM diagnoses codes, Current Procedural Terminology (CPT) or Healthcare Common Procedure Coding System (HCPCS) procedure codes, or National Drug Code (NDC) for prescription therapies.

### Statistical analysis

The distributions of demographic variables and baseline cardiovascular risk factors by treatment group were described. Propensity scores were estimated using multivariable logistic regression. The overlap of the propensity score distributions in each cohort was visualized to assess possible positivity violations and 1% trimming was applied. Differences in baseline patient characteristics between the treatment groups were assessed in the original (unweighted) cohorts and the IPTW cohorts using standardized mean differences (SMD). Any variable with an absolute value of the SMD greater than 0.1 (10%) was considered a clinically significant difference. Assessment of covariate balance in each cohort and subcohort was completed in the absence of any information on outcomes in the data set.

Cumulative risks at each of the three time points were calculated. Adjusted RRs and differences (with 95% CIs) were estimated, using a semiparametric cumulative risk estimator. Analyses were performed using R version 3.6 (R Foundation for Statistical Computing, Vienna, Austria; https://www.r-project.org/) and the causalRisk package of functions (https://docs.novisci.com/causalRisk/) and SAS version 9.14 (SAS Institute, Inc., Cary, NC, USA).

### Subcohorts

Denosumab, unlike zoledronic acid, is not contraindicated in patients with creatinine clearance less than 35 mL/min or in those with evidence of acute renal impairment. Therefore, we expected that patients treated with denosumab would have a higher prevalence of renal impairment than those treated with zoledronic acid. Because renal insufficiency is a strong indicator of cardiovascular risk, we expected that denosumab‐treated patients may have a higher incidence of cardiovascular events due to co‐occurring renal insufficiency, regardless of any drug‐exposure effect. To address this potential bias, subgroup analyses were prespecified for six stages of chronic kidney disease (CKD). However, due to the low number of events and small sample size in the CKD subcohorts, we assessed only the subcohort with no CKD.

### Sensitivity analyses

Due to differing pathophysiology and preponderance of ischemic versus hemorrhagic stroke, the incidence of stroke was further characterized by performing a sensitivity analysis restricting stroke events to ischemic stroke events.^[^ ^25^, ^26^ ^]^

Primary analyses in the Optum data included death among the censoring variables. Death may be a competing risk in these analyses. To address this potential bias, a competing risk analysis was performed in Optum data for the outcomes of MI, stroke, and the composite MI/stroke outcome using an appropriate competing risk model that addresses confounding and informative censoring.^[^ ^27^ ^]^

A post hoc intention‐to‐treat (ITT) sensitivity analysis, ignoring treatment switches and discontinuations, was conducted to address concerns of differential follow‐up between treatment groups.

Some risk factors for cardiovascular disease, such as body weight index and smoking, are not well captured in claims data. Quantitative bias analyses were conducted to assess the potential impact of unmeasured confounding.^[^ ^28^ ^]^ Quantitative bias analyses provide corrected point estimates and 95% CIs for the treatment effect over a range of potential values of the relative risk for an unmeasured confounder with exposure and the relative risk for an unmeasured confounder with the outcome.

Statistical analyses in the final analytical data sets were conducted by two independent analysts and cross‐checked for quality assurance.

## Results

### Study population

During the study period (October 1, 2010, to June 30, 2019), 96,611 patients aged ≥55 years in the MarketScan database and 73,127 in the Optum database met all study inclusion/exclusion criteria (Table S1); the weighted sample size after 1% propensity score trimming was 94,677 in MarketScan and 71,663 in Optum.

The median study follow‐up time for all patients across different outcomes was 384–386 days for MarketScan and 349–350 days for Optum. The median study follow‐up time was longer for patients in the zoledronic acid group (426 days) than for those in the denosumab group (247–260 days) (Table S2).

Overall, >90% of the patients in each cohort was female. The mean age of the patients in Optum was slightly higher (71 in the zoledronic acid group and 73 in the denosumab group) than those in MarketScan (68 in the zoledronic acid group and 69 in the denosumab group). In the unweighted analysis, most of baseline characteristics were balanced except for region, lower percentage of males, higher mean age, later mean year of cohort entry, and a higher percentage of a history of CKD in denosumab users (|SMD| ≥ 0.10) (Tables 1 and 2). In addition, in Optum there were treatment group imbalances in race (Table 2).

**Table 1:** Unweighted and Weighted (1% Trimmed) Baseline Characteristics for MarketScan

Characteristic	Unweighted			Weighted		
	Zoledronic acid (*n* = 40,543)	Denosumab (*n* = 56,068)	SMD	Zoledronic acid (*n* = 39,531)	Denosumab (*n* = 55,146)	SMD
Sex						
Female	37,247 (91.87)	53,134 (94.77)	0.12	92,994 (94.11)	87,337 (93.96)	0.01
Male	3296 (8.13)	2934 (5.23)		5819 (5.89)	5612 (6.04)	
Age, years, mean (SD)	67.88 (9.50)	69.48 (10.17)	−0.16	68.48 (9.67)	68.66 (9.97)	−0.02
Age groups, years						
55–64	19,673 (48.52)	24,213 (43.19)	0.13	45,908 (46.46)	43,127 (46.40)	0.01
65–74	10,148 (25.03)	13,737 (24.50)		24,582 (24.88)	22,687 (24.41)	
75+	10,722 (26.45)	18,118 (32.31)		28,324 (28.66)	27,135 (29.19)	
Region						
Northeast	6908 (17.04)	7539 (13.45)	0.26	14,765 (14.94)	13,894 (14.95)	0.02
Midwest	10,329 (25.48)	10,225 (18.24)		20,510 (20.76)	19,517 (21.00)	
South	12,553 (30.96)	19,453 (34.70)		32,047 (32.43)	30,667 (32.99)	
West	5778 (14.25)	8154 (14.54)		14,891 (15.07)	13,583 (14.61)	
Unknown	4975 (12.27)	10,697 (19.08)		16,599 (16.80)	15,288 (16.45)	
Charlson comorbidity index						
0	29,930 (73.82)	40,844 (72.85)	0.05	73,275 (74.16)	68,766 (73.98)	<0.01
1	7111 (17.54)	9579 (17.08)		16,646 (16.85)	15,744 (16.94)	
2	2094 (5.16)	3322 (5.92)		5289 (5.35)	5051 (5.43)	
3+	1408 (3.47)	2323 (4.14)		3603 (3.65)	3388 (3.65)	
Atrial fibrillation	1872 (4.62)	3370 (6.01)	0.06	4958 (5.02)	4866 (5.24)	0.01
Angina	403 (0.99)	573 (1.02)	<0.01	909 (0.92)	900 (0.97)	<0.01
Asthma	2961 (7.30)	4251 (7.58)	0.01	7336 (7.42)	6889 (7.41)	<0.01
BMD test	24,532 (60.51)	31,411 (56.02)	0.09	57,866 (58.56)	53,921 (58.01)	0.01
CABG or PCI	77 (0.19)	73 (0.13)	0.01	146 (0.15)	138 (0.15)	<0.01
CKD (any)	1719 (4.24)	4838 (8.63)	0.18	6321 (6.40)	5802 (6.24)	0.01
CKD stage						
1 or 2	111 (0.27)	239 (0.43)	0.20	369 (0.37)	344 (0.37)	0.02
3	480 (1.18)	1787 (3.19)		2114 (2.14)	1961 (2.11)	
4	29 (0.07)	460 (0.82)		139 (0.14)	151 (0.16)	
5	4 (0.01)	32 (0.06)		10 (0.01)	20 (0.02)	
Unknown or unspecified	1020 (2.52)	2029 (3.62)		3288 (3.33)	3004 (3.23)	
ESRD	75 (0.18)	291 (0.52)		401 (0.41)	322 (0.35)	
No CKD	38,824 (95.76)	51,230 (91.37)		92,493 (93.60)	87,147 (93.76)	
COPD	3383 (8.34)	4574 (8.16)	0.01	7743 (7.84)	7458 (8.02)	0.01
Conduction disorder	215 (0.53)	440 (0.78)	0.03	666 (0.67)	624 (0.67)	<0.01
Dementia	463 (1.14)	828 (1.48)	0.03	1218 (1.23)	1192 (1.28)	<0.01
Depressive disorder	2348 (5.79)	3055 (5.45)	0.01	5687 (5.76)	5292 (5.69)	<0.01
Hypercholesterolemia	3563 (8.79)	5502 (9.81)	0.04	9137 (9.25)	8658 (9.31)	<0.01
Heart failure	1238 (3.05)	2262 (4.03)	0.05	3374 (3.41)	3220 (3.46)	<0.01
Hypertension	15,706 (38.74)	24,003 (42.81)	0.08	39,659 (40.13)	37,708 (40.57)	0.01
Inflammatory arthritis	1605 (3.96)	1898 (3.39)	0.03	3554 (3.60)	3337 (3.59)	<0.01
Obesity	597 (1.47)	915 (1.63)	0.01	1603 (1.62)	1476 (1.59)	<0.01
Osteoporosis diagnosis	17,228 (42.49)	25,707 (45.85)	0.07	45,305 (45.85)	41,831 (45.00)	0.02
Fragility fracture	3242 (8.00)	5406 (9.64)	0.06	8515 (8.62)	8219 (8.84)	0.01
Peripheral vascular disease	1197 (2.95)	2135 (3.81)	0.05	3247 (3.29)	3111 (3.35)	<0.01
Pneumonia	1035 (2.55)	1311 (2.34)	0.01	2258 (2.29)	2146 (2.31)	<0.01
Medications						
Androgen replacement	208 (0.51)	230 (0.41)	0.02	427 (0.43)	416 (0.45)	<0.01
Antianginal nitrate	1068 (2.63)	1483 (2.65)	<0.01	2451 (2.48)	2376 (2.56)	<0.01
Antianginal ranolazine	88 (0.22)	159 (0.28)	0.01	264 (0.27)	238 (0.26)	<0.01
Antiarrhythmic	489 (1.21)	960 (1.71)	0.04	1443 (1.46)	1357 (1.46)	<0.01
Anticoagulant	2133 (5.26)	3590 (6.40)	0.05	5456 (5.52)	5342 (5.75)	0.01
Anticonvulsant	6064 (14.96)	8783 (15.66)	0.02	15,374 (15.56)	14,386 (15.48)	<0.01
Antidepressant	11,497 (28.36)	15,663 (27.94)	0.01	28,084 (28.42)	26,371 (28.37)	<0.01
Antidiabetic insulin	976 (2.41)	1576 (2.81)	0.03	2683 (2.72)	2413 (2.60)	0.01
Antidiabetic non‐insulin	2635 (6.50)	4080 (7.28)	0.03	6820 (6.90)	6418 (6.90)	<0.01
Antihypertensive	10,673 (26.33)	17,202 (30.68)	0.10	28,062 (28.40)	26,551 (28.57)	<0.01
Antiparkinsonian	1204 (2.97)	1548 (2.76)	0.01	2812 (2.85)	2643 (2.84)	<0.01
Antithrombotic	1360 (3.35)	2126 (3.79)	0.02	3428 (3.47)	3301 (3.55)	<0.01
Antipsychotic	1193 (2.94)	1536 (2.74)	0.01	2777 (2.81)	2620 (2.82)	<0.01
Benzodiazepine	6111 (15.07)	9037 (16.12)	0.03	15,613 (15.80)	14,623 (15.73)	<0.01
Digoxin	157 (0.39)	352 (0.63)	0.03	513 (0.52)	483 (0.52)	<0.01
Estrogen replacement	2052 (5.06)	2535 (4.52)	0.03	4713 (4.77)	4458 (4.80)	<0.01
Flu vaccine	13,961 (34.44)	18,512 (33.02)	0.03	33,566 (33.97)	31,267 (33.64)	0.01
Lipid lowering	13,983 (34.49)	21,804 (38.89)	0.09	36,306 (36.74)	34,325 (36.93)	<0.01
NSAID or Cox‐2 inhibitor	9293 (22.92)	13,732 (24.49)	0.04	23,551 (23.83)	22,366 (24.06)	0.01
Opioid	15,812 (39.00)	22,293 (39.76)	0.02	38,839 (39.31)	36,705 (39.49)	<0.01
Oral bisphosphonate	9120 (22.49)	14,129 (25.20)	0.06	24,083 (24.37)	22,422 (24.12)	0.01
Proton pump inhibitor	11,003 (27.14)	14,784 (26.37)	0.02	26,197 (26.51)	24,796 (26.68)	<0.01
Schizophrenia	207 (0.51)	274 (0.49)	<0.01	469 (0.47)	443 (0.48)	<0.01
Sepsis or septicemia	380 (0.94)	522 (0.93)	<0.01	850 (0.86)	826 (0.89)	<0.01
Sleep apnea	1767 (4.36)	2244 (4.00)	0.02	4108 (4.16)	3834 (4.13)	<0.01
Smoking	1071 (2.64)	1298 (2.32)	0.02	2450 (2.48)	2287 (2.46)	<0.01
Transient ischemic attack	281 (0.69)	442 (0.79)	0.01	733 (0.74)	682 (0.73)	<0.01
Type 2 diabetes	4495 (11.09)	6490 (11.58)	0.02	10,900 (11.03)	10,390 (11.18)	<0.01
Urinary incontinence	694 (1.71)	1022 (1.82)	0.01	1773 (1.79)	1659 (1.78)	<0.01
At least one outpatient visit	40,464 (99.81)	55,860 (99.63)	0.03	98,614 (99.80)	92,609 (99.63)	0.03
Outpatient visit count, mean (SD)	27.37 (22.14)	27.37 (23.21)	<0.01	27.07 (21.64)	27.18 (23.09)	<0.01
At least one inpatient visit	5862 (14.46)	7889 (14.07)	0.01	13,252 (13.41)	12,851 (13.83)	0.01
Inpatient visit count, mean (SD)	0.19 (0.55)	0.18 (0.50)	0.02	0.17 (0.50)	0.18 (0.50)	−0.01
At least one ER visit	12,040 (29.70)	16,676 (29.74)	<0.01	29,073 (29.42)	27,160 (29.22)	<0.01
ER visit count, mean (SD)	0.49 (1.05)	0.50 (1.06)	<0.01	0.49 (1.04)	0.49 (1.06)	<0.01
Year of cohort entry, mean (SD)	2012.96 (2.29)	2014.55 (2.16)	−0.71	2014.20 (2.68)	2014.02 (2.08)	0.08

*Note*: Values are *n* (%) except where mean (SD) is indicated in the variable label.

Abbreviations: BMD = bone mineral density; CABG = coronary artery bypass graft; CKD = chronic kidney disease; COPD = chronic obstructive pulmonary disease; ER = emergency room; ESRD = end‐stage renal disease; NSAID = non‐steroidal anti‐inflammatory drug; PCI = percutaneous coronary intervention; SMD = standardized mean difference.

**Table 2:** Unweighted and Weighted (1% Trimmed) Baseline Characteristics for Optum

	Unweighted			Weighted		
Characteristic	Zoledronic acid (*n* = 23,576)	Denosumab (*n* = 49,551)	SMD	Zoledronic acid (*n* = 22,943)	Denosumab (*n* = 48,720)	SMD
Sex						
Female	21,590 (91.58)	46,687 (94.22)	0.10	68,125 (93.75)	66,870 (93.66)	<0.01
Male	1986 (8.42)	2864 (5.78)		4541 (6.25)	4526 (6.34)	
Race						
Asian	620 (2.63)	3090 (6.24)	0.23	3663 (5.04)	3544 (4.96)	0.01
Black	1601 (6.79)	2981 (6.02)		4420 (6.08)	4430 (6.20)	
Unknown	3591 (15.23)	9951 (20.08)		13,499 (18.58)	13,289 (18.61)	
White	17,764 (75.35)	33,529 (67.67)		51,084 (70.30)	50,134 (70.22)	
Age, years, mean (SD)	71.54 (8.33)	73.43 (8.26)	−0.23	72.82 (8.19)	72.84 (8.35)	<0.01
Age group, years						
55–64	5315 (22.54)	7738 (15.62)	0.20	12,522 (17.23)	12,722 (17.82)	0.02
65–74	9161 (38.86)	18,929 (38.20)		28,257 (38.89)	27,472 (38.48)	
75+	9100 (38.60)	22,884 (46.18)		31,886 (43.88)	31,203 (43.70)	
Region						
Northeast	2492 (10.57)	5020 (10.13)	0.19	7434 (10.23)	7376 (10.33)	0.01
Midwest	6647 (28.19)	10,175 (20.53)		16,527 (22.74)	16,250 (22.76)	
South	9192 (38.99)	21,336 (43.06)		30,264 (41.65)	29,879 (41.85)	
West	5228 (22.18)	12,985 (26.21)		18,387 (25.30)	17,840 (24.99)	
Unknown	17 (0.07)	35 (0.07)		54 (0.07)	52 (0.07)	
Charlson comorbidity index						
0	15,250 (64.68)	31,077 (62.72)	0.08	46,496 (63.99)	45,718 (64.03)	<0.01
1	5018 (21.28)	10,219 (20.62)		15,039 (20.70)	14,765 (20.68)	
2	1908 (8.09)	4536 (9.15)		6282 (8.65)	6174 (8.65)	
3+	1400 (5.94)	3719 (7.51)		4848 (6.67)	4740 (6.64)	
Atrial fibrillation	1605 (6.81)	3797 (7.66)	0.03	5408 (7.44)	5220 (7.31)	0.01
Angina	249 (1.06)	629 (1.27)	0.02	846 (1.16)	828 (1.16)	<0.01
Asthma	2592 (10.99)	5824 (11.75)	0.02	8429 (11.60)	8175 (11.45)	<0.01
BMD test	17,923 (76.02)	38,250 (77.19)	0.03	56,121 (77.23)	54,938 (76.95)	0.01
CABG or PCI	31 (0.13)	49 (0.10)	0.01	83 (0.11)	73 (0.10)	<0.01
CKD (any)	2281 (9.68)	7904 (15.95)	0.19	9797 (13.48)	9484 (13.28)	0.01
CKD stage						
1 or 2	256 (1.09)	638 (1.29)	0.23	948 (1.31)	898 (1.26)	0.01
3	866 (3.67)	3548 (7.16)		4489 (6.18)	4334 (6.07)	
4	37 (0.16)	693 (1.40)		191 (0.26)	192 (0.27)	
5	2 (0.01)	44 (0.09)		8 (0.01)	8 (0.01)	
Unknown or unspecified	1056 (4.48)	2715 (5.48)		3827 (5.27)	3737 (5.23)	
ESRD	64 (0.27)	266 (0.54)		333 (0.46)	314 (0.44)	
No CKD	21,295 (90.32)	41,647 (84.05)		62,868 (86.52)	61,913 (86.72)	
COPD	2872 (12.18)	6013 (12.13)	<0.01	8774 (12.07)	8579 (12.02)	<0.01
Conduction disorder	350 (1.48)	849 (1.71)	0.02	1189 (1.64)	1165 (1.63)	<0.01
Dementia	589 (2.50)	1603 (3.24)	0.04	2126 (2.93)	2121 (2.97)	<0.01
Depressive disorder	2183 (9.26)	4633 (9.35)	<0.01	6850 (9.43)	6627 (9.28)	<0.01
Hypercholesterolemia	3019 (12.81)	6456 (13.03)	0.01	9199 (12.66)	9182 (12.86)	0.01
Heart failure	1122 (4.76)	2869 (5.79)	0.05	3808 (5.24)	3732 (5.23)	<0.01
Hypertension	12,332 (52.31)	27,712 (55.93)	0.07	39,538 (54.41)	38,841 (54.40)	<0.01
Inflammatory arthritis	1157 (4.91)	1949 (3.93)	0.05	3071 (4.23)	2985 (4.18)	<0.01
Obesity	768 (3.26)	1807 (3.65)	0.02	2576 (3.54)	2490 (3.49)	<0.01
Osteoporosis diagnosis	12,765 (54.14)	27,406 (55.31)	0.02	40,559 (55.82)	39,393 (55.17)	0.01
Fragility fracture	2330 (9.88)	5516 (11.13)	0.04	7768 (10.69)	7625 (10.68)	<0.01
Peripheral vascular disease	1336 (5.67)	3679 (7.42)	0.07	5025 (6.91)	4829 (6.76)	0.01
Pneumonia	742 (3.15)	1401 (2.83)	0.02	2116 (2.91)	2035 (2.85)	<0.01
Medications						
Androgen replacement	138 (0.59)	156 (0.31)	0.04	268 (0.37)	266 (0.37)	<0.01
Antianginal nitrate	639 (2.71)	1251 (2.52)	0.01	1793 (2.47)	1784 (2.50)	<0.01
Antianginal ranolazine	57 (0.24)	148 (0.30)	0.01	178 (0.24)	189 (0.27)	<0.01
Antiarrhythmic	353 (1.50)	908 (1.83)	0.03	1263 (1.74)	1214 (1.70)	<0.01
Anticoagulant	1572 (6.67)	3580 (7.22)	0.02	5125 (7.05)	4977 (6.97)	<0.01
Anticonvulsant	4351 (18.46)	9041 (18.25)	0.01	13,268 (18.26)	12,996 (18.20)	<0.01
Antidepressant	7465 (31.66)	14,482 (29.23)	0.05	21,724 (29.90)	21,364 (29.92)	<0.01
Antidiabetic insulin	683 (2.90)	1619 (3.27)	0.02	2140 (2.94)	2138 (2.99)	<0.01
Antidiabetic non‐insulin	2039 (8.65)	4857 (9.80)	0.04	6684 (9.20)	6620 (9.27)	<0.01
Antihypertensive	7875 (33.40)	18,547 (37.43)	0.08	25,928 (35.68)	25,651 (35.93)	0.01
Antiparkinsonian	852 (3.61)	1650 (3.33)	0.02	2486 (3.42)	2431 (3.40)	<0.01
Antithrombotic	844 (3.58)	2127 (4.29)	0.04	2865 (3.94)	2839 (3.98)	<0.01
Antipsychotic	825 (3.50)	1543 (3.11)	0.02	2358 (3.24)	2301 (3.22)	<0.01
Benzodiazepine	3205 (13.59)	7252 (14.64)	0.03	10,576 (14.55)	10,260 (14.37)	0.01
Digoxin	100 (0.42)	288 (0.58)	0.02	360 (0.49)	372 (0.52)	<0.01
Estrogen replacement	804 (3.41)	1230 (2.48)	0.05	2010 (2.77)	1972 (2.76)	<0.01
Flu vaccine	12,045 (51.09)	24,563 (49.57)	0.03	36,260 (49.90)	35,669 (49.96)	<0.01
Lipid lowering	9523 (40.39)	21,450 (43.29)	0.06	30,596 (42.11)	30,118 (42.18)	<0.01
NSAID or Cox‐2 inhibitor	6066 (25.73)	12,905 (26.04)	0.01	18,723 (25.77)	18,544 (25.97)	<0.01
Opioid	9894 (41.97)	19,779 (39.92)	0.04	29,383 (40.44)	28,781 (40.31)	<0.01
Oral bisphosphonate	5779 (24.51)	13,609 (27.46)	0.07	19,093 (26.27)	18,943 (26.53)	0.01
Proton pump inhibitor	7518 (31.89)	14,722 (29.71)	0.05	22,034 (30.32)	21,577 (30.22)	<0.01
Schizophrenia	196 (0.83)	318 (0.64)	0.02	506 (0.70)	491 (0.69)	<0.01
Sepsis or septicemia	305 (1.29)	633 (1.28)	<0.01	940 (1.29)	888 (1.24)	<0.01
Sleep apnea	1258 (5.34)	2333 (4.71)	0.03	3549 (4.88)	3447 (4.83)	<0.01
Smoking	1049 (4.45)	2073 (4.18)	0.01	3112 (4.28)	3044 (4.26)	<0.01
Transient ischemic attack	189 (0.80)	429 (0.87)	0.01	578 (0.80)	593 (0.83)	<0.01
Type 2 diabetes	3435 (14.57)	7965 (16.07)	0.04	11,018 (15.16)	10,891 (15.25)	<0.01
Urinary incontinence	641 (2.72)	1518 (3.06)	0.02	2153 (2.96)	2110 (2.96)	<0.01
At least one outpatient visit	23,509 (99.72)	48,826 (98.54)	0.13	72,415 (99.66)	70,423 (98.64)	0.11
Outpatient visit count, mean (SD)	28.56 (23.11)	28.14 (24.17)	0.02	28.12 (22.37)	28.08 (24.10)	<0.01
At least one inpatient visit	3673 (15.58)	7358 (14.85)	0.02	10,717 (14.75)	10,624 (14.88)	<0.01
Inpatient visit count, mean (SD)	0.22 (0.61)	0.20 (0.56)	0.03	0.20 (0.56)	0.20 (0.56)	<0.01
At least one ER visit	7509 (31.85)	14,957 (30.19)	0.04	22,628 (31.14)	21,599 (30.25)	0.02
ER visit count, mean (SD)	0.55 (1.22)	0.52 (1.15)	0.02	0.53 (1.09)	0.53 (1.17)	<0.01
Year of cohort entry, mean (SD)	2014.67 (2.81)	2015.92 (2.26)	−0.49	2015.69 (2.69)	2015.59 (2.33)	0.04

*Note*: Values are *n* (%) except where mean (SD) is indicated in the variable label.

Abbreviations: BMD = bone mineral density; CABG = coronary artery bypass graft; CKD = chronic kidney disease; COPD = chronic obstructive pulmonary disease; ER = emergency room; ESRD = end‐stage renal disease, NSAID = non‐steroidal anti‐inflammatory drug; PCI = percutaneous coronary intervention; SMD = standardized mean difference.

After IPTW adjustment and with 1% propensity score trimming, all baseline characteristics were comparable between denosumab and zoledronic acid treatment groups with |SMD| ≤0.10 for both MarketScan and Optum databases (Tables 1 and 2).

### Risks, RRs, and RDs

In MarketScan and Optum, respectively, the numbers of stroke events were 109 and 99 (zoledronic acid group), 147 and 150 (denosumab group); the numbers of MIs were 151 and 103 (zoledronic acid group) and 232 and 229 (denosumab group). Cumulative risks of individual primary outcomes (MI or stroke) in both treatment groups were between 0.11% and 0.23% at 6 months in both databases. These risks approximately doubled at 12 months (0.23% to 0.44%). Cumulative risks at 36 months were between 0.51% and 1.39%.

**Fig. 1:**
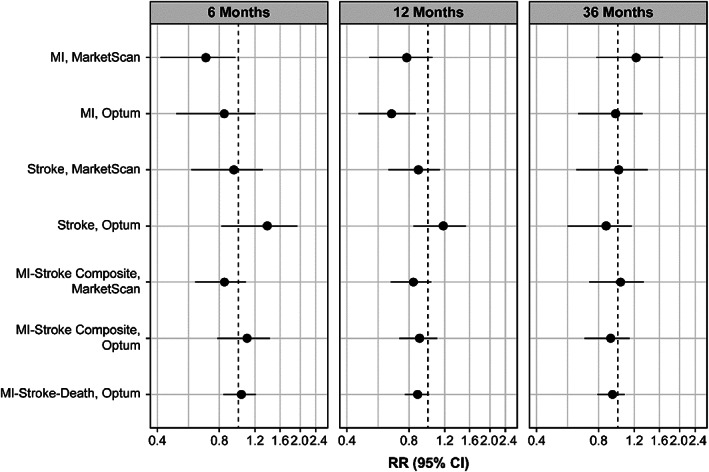
Summary forest plot for all outcomes in each database, full cohorts. RR axis is presented on a log scale. RR < 1.0 indicates a higher risk in the zoledronic acid group; RR >1.0 indicates a higher risk in the denosumab group. All results are from inverse probability of treatment and censoring weighted estimation functions. Propensity score trimming by 1% was applied. MI = myocardial infarction; RR = risk ratio.

At 36 months, RR estimates (zoledronic acid referent group) for MI were 1.22 (95% CI, 0.77–1.66) and 0.97 (95% CI, 0.63–1.32) and for stroke were 1.00 (95% CI, 0.61–1.40) and 0.87 (95% CI, 0.56–1.17) in MarketScan and Optum, respectively (Fig. 1). Nearly all treatment associations across the other time periods and outcomes also had 95% CIs including the null value, with the exceptions for two MI associations that occurred within 1 year of initiating treatment: (i) At 6 months, the RR for MI in MarketScan indicated a lower risk in the denosumab treatment group (RR = 0.69; 95% CI, 0.41–0.98) than in the zoledronic acid treatment group. However, the RD at this time point was small (RD = −0.05; 95% CI, −0.11 to 0.01), reflecting low cumulative risks (0.16% in zoledronic acid treatment group versus 0.11% in denosumab treatment group). (ii) At 12 months, the RR for MI in Optum indicated a lower risk in the denosumab treatment group than in the zoledronic acid treatment group (RR = 0.66; 95% CI, 0.45–0.87). The RD at this time point was slightly larger (RD = −0.14; 95% CI, −0.26 to −0.03) and cumulative risks were 0.42% in zoledronic acid treatment group and 0.28% in the denosumab treatment group (Table S3 and Fig. S1).

### Sensitivity analyses

Among patients with no CKD, the cumulative risk of cardiovascular events at 36 months after treatment initiation was similar to that of the full cohort for each outcome in both databases (Tables 3 and S4).

**Table 3:** Sensitivity Analyses—Cumulative Risk by Treatment Group and Adjusted Risk Ratio at 36 Months for Each Outcome

Myocardial infarction			Stroke		
Treatment	CR per 100 (95% CI)	RR (95% CI)	Treatment	CR per 100 (95% CI)	RR (95% CI)
**MarketScan**			**MarketScan**		
**No CKD**			**No CKD**		
Zoledronic acid	0.49 (0.34–0.65)		Zoledronic acid	1.10 (0.68–1.52)	
Denosumab	0.57 (0.42–0.72)	1.16 (0.68–1.64)	Denosumab	0.98 (0.77–1.20)	0.89 (0.50–1.28)
**ITT analysis**			**ITT analysis**		
Zoledronic acid	0.94 (0.77–1.11)		Zoledronic acid	1.24 (1.04–1.44)	
Denosumab	0.89 (0.76–1.01)	0.94 (0.72–1.16)	Denosumab	1.21 (1.06–1.36)	0.98 (0.78–1.17)
			**Ischemic stroke**		
			Zoledronic acid	1.00 (0.63–1.36)	
			Denosumab	1.00 (0.77–1.23)	1.00 (0.57–1.44)
**Optum**			**Optum**		
**No CKD**			**No CKD**		
Zoledronic acid	0.95 (0.66–1.24)		Zoledronic acid	1.30 (0.80–1.79)	
Denosumab	0.80 (0.62–0.98)	0.84 (0.52–1.17)	Denosumab	1.08 (0.91–1.25)	0.83 (0.49–1.18)
**ITT analysis**			**ITT analysis**		
Zoledronic acid	1.26 (1.08–1.44)		Zoledronic acid	1.75 (1.53–1.97)	
Denosumab	1.18 (1.04–1.33)	0.94 (0.76–1.11)	Denosumab	1.58 (1.45–1.71)	0.90 (0.77–1.04)
			**Ischemic stroke**		
			Zoledronic acid	1.30 (0.85–1.76)	
			Denosumab	1.04 (0.89–1.20)	0.80 (0.50–1.10)
**With death modeled as a competing risk**			**With death modeled as a competing risk**		
Zoledronic acid	0.97 (0.70–1.24)		Zoledronic acid	1.29 (0.87–1.70)	
Denosumab	1.02 (0.83–1.21)	1.06 (0.70–1.41)	Denosumab	1.33 (1.17–1.49)	1.03 (0.68–1.39)

*Note*: RR < 1.0 indicates a higher risk in the zoledronic acid group; RR > 1.0 indicates a higher risk in the denosumab group. All results are from inverse probability of treatment and censoring weighted estimation functions. Propensity score trimming by 1% was applied.

Abbreviation: CKD = chronic kidney disease; CR = cumulative risk; ITT = intention‐to‐treat; RR = risk ratio.

At 36 months, for ischemic stroke analysis, all 95% CIs for effect estimates included the null, and MarketScan cumulative risk percentages were identical between the two treatment groups. In Optum, the RR of ischemic stroke was somewhat lower (0.80) than overall (ischemic and hemorrhagic) stroke RR (0.87), indicating a lower risk in the denosumab treatment group. However, 95% CIs included the null value for both outcomes (Tables 3 and S5).

All primary results for Optum modeled death as a censoring event so that the specification of death would be comparable to that in MarketScan, in which death was indistinguishable from other reasons for disenrollment. The results of a competing risk analysis were consistent with the findings of the primary analysis (Tables 3 and S6).

Consistent with the primary analysis, null findings were found in the post hoc ITT analyses for MI, stroke, and MI‐stroke composite outcomes for MarketScan and Optum (Tables 3 and S7).

To further assess the potential for residual confounding from unmeasured confounders, quantitative bias analysis was performed for each outcome in each cohort (Tables S8–S11). Literature suggests that an RR of 2 is a reasonable assumption for the potential magnitude of association between smoking and body mass index (BMI) with study outcomes. Assuming the RR for smoking or BMI and the outcome is 2, denosumab users would need to be two or more times more likely to be smokers then zoledronic acid users to change the null results of our primary analysis. Because there is no evidence that smoking or BMI are strongly related to treatment choice, bias by unmeasured confounding is unlikely. In the analysis of stroke in Optum, denosumab users would need to be 1.5 times more likely to be smokers than zoledronic acid users to change the null results of our primary analysis, suggesting that the analyses of stroke could be somewhat more susceptible to bias from unmeasured confounding. In this case, the RR of stroke for denosumab (versus zoledronic acid) would move from the observed RR of 0.87 (95% CI, 0.56–1.17) to an RR of 0.72 (95% CI, 0.47–0.98), which ultimately would not impact the interpretation of study results.

## Discussion

At 36 months, the adjusted cumulative risks of MI, stroke, composite cardiovascular disease outcome, and the composite outcome including death were similar between treatment groups in both databases. Nearly all treatment associations across the earlier time frames had 95% CIs that included the null value. Exceptions were for two MI associations occurring within 1 year of initiating medication. This slightly lower risk of MI at 6 and 12 months observed in the denosumab users translated to a minor reduction in absolute risk and therefore was not considered to be clinically meaningful.

Our results are supported by both meta‐analyses of randomized clinical trials and previous observational research. Three meta‐analyses have assessed the association of anti‐RANKL on cardiovascular events and included studies published until 2019, including two analyses conducted in postmenopausal women. One meta‐analysis assessing randomized controlled trials (RCTs) of denosumab versus a heterogeneous control group (active comparators or placebo) including 11 studies and 13,615 participants found no association for denosumab and composite or individual cardiovascular events.^[^ ^29^ ^]^ The other two studies also reported no significant difference between placebo and denosumab in cardiovascular adverse events.^[^ ^30^, ^31^ ^]^ However, one study reported an increase in cardiovascular adverse events for denosumab compared with bisphosphonates. The authors surmise that these findings intimate a relative suppression of events in the bisphosphonate‐treated women.^[^ ^31^ ^]^ The meta‐analyses of RCTs are limited in that cardiac adverse events were not the primary outcome of the included studies.

Previous observational research conducted using new user, propensity score matching designs have also evaluated this association and reported similar findings. The first study using 2009 to 2013 US commercial insurance claims data (Optum) reported an incidence rate ratio of denosumab users relative to zoledronic acid users (zoledronic acid reference group) to be 1.24 (95% CI, 0.25–6.14) for MI and 0.31 (95% CI, 0.03–2.77) for stroke.^[^ ^19^ ^]^ This study included 2467 patients in each treatment group, but only identified a total of six MI events (three events each exposure group) and five stroke events (one event in the denosumab group and four in the zoledronic acid group) over a 1‐year period. A second study was designed to assess incident diagnosis of atrial fibrillation over 1 year, but included stroke/transient ischemic attack as a secondary possible downstream safety outcome. In the osteoporosis cohort they reported, 69 events of stroke/transient ischemic attack in the zoledronic acid group and 60 events in the denosumab group (reference group) with a hazard ratio of 1.15 (95% CI, 0.81–1.62).^[^ ^32^ ^]^ A third study^[^ ^33^ ^]^ was conducted in one large academic medical center in Taiwan and compared denosumab and alendronate on cardiovascular outcomes for up to 5 years in osteoporotic patients using 2005–2017 data and reported no significant differences in cardiovascular events.

Our study has several significant strengths. It was conducted in a real‐world population of patients being treated for osteoporosis. The study had a large sample size, with up to 3 years of follow‐up, capturing a sufficient number of cardiovascular and cerebrovascular events to generate precise risk estimates. Confounding by indication was addressed by state‐of‐the‐art IPTW and censoring weighted adjustment methods and followed a staged analytic pipeline designed to guard against biased analytic decisions. The results of the study were replicated across two well‐characterized US data systems. In addition, zoledronic acid was shown to be an appropriate comparator arm for denosumab in a negative control outcome study that assessed the comparability of osteoporosis treatments after adjustment for measured confounders.^[^ ^34^ ^]^

This study also had limitations. Renal insufficiency may be a strong indicator of cardiovascular risk. Although CKD was included in IPTW adjustment and the CKD covariate was balanced between the treatment groups, estimates could still be influenced by residual confounding biasing the results against the denosumab group. The less severe CKD stages 1 and 2 cases could have been underreported through ICD codes by the physician. However, the association between CKD stages 1 and 2 and cardiovascular outcomes is probably weaker than the association of higher stages of CKD and cardiovascular outcomes. In this study, the results from the subcohort with no CKD were consistent with those of the primary analysis.

Residual confounding may remain due to missing or misclassified variables. To address confounding in general, the cumulative risk of each outcome was estimated using IPTW and censoring weighted estimation functions. Balance was achieved in the measured covariates between the different treatment groups after IPTW. The smoking and obesity covariates included in the propensity score had an |SMD| of <0.10 in the unweighted tables, which indicated a balance between the denosumab and zoledronic acid cohorts. However, these variables may have been incompletely captured in claims data and it could result in residual bias. Other variables that were included in the propensity score model (sex, chronic obstructive pulmonary disease, asthma, diabetes, depressive disorder) could also partially account for any residual differences from incompletely captured BMI and smoking among the treatment groups.^[^ ^35^ ^]^ Similarly, Tanko and colleagues^[^ ^36^ ^]^ (2005) noted that an increased risk of cardiovascular events was associated with severity of osteoporosis, as indicated by prior vertebral fracture. Although we included osteoporosis diagnosis and fragility fracture covariates, which included clinical vertebral fracture and were balanced after IPTW, we could not assess whether history of morphologic vertebral fracture differed between treatment groups.

To further assess the potential for residual confounding from unmeasured confounders, quantitative bias analysis was performed. The results indicated that it is unlikely that our findings are biased due to unmeasured confounding.

Because the main outcome of the study was hospitalization for MI and stroke, death could introduce a competing risk in the study analysis. The magnitude and proportionality of the death rates in the denosumab and zoledronic acid cohorts were assessed. Only a small percentage of patients were censored for death and the percentage was nondifferential. This provides reassurance that death as a competing risk would have a minimal impact on the results. Comparing the primary results from Optum (not accounting for death as a competing risk) to the sensitivity results (accounting for competing risk) also provides insight into the extent of potential bias caused by competing risks. In this study, the results from the competing risk analysis were consistent with those of the primary analysis. The competing risk may have even less influence in the younger MarketScan population where no mortality information is available.

Because loss to follow‐up included treatment switch and discontinuation in the primary analysis, we performed a post hoc ITT analysis that included these patients and results were similar to the initial findings.

The median follow‐up in our study was 386 days in MarketScan and 350 days Optum (Table S2). Approximately 6% of the cohorts reached the end of the 3‐year follow‐up period (Table S2). To address potentially informative censoring, weighted estimation functions were used. Censoring weights were applied to the uncensored population to weight them up to resemble the overall uncensored population. However, our 3‐year estimates were more heavily weighted from information in the earlier timeframe. The databases that were used contain the healthcare experience of commercially insured employees and their dependents which may be a younger population at lower risk, enrollment may be limited by patients changing plans and at age 65 years or older moving to Medicare and may not be generalizable to the uninsured population. These results may not represent men because men only account for 6% of the overall cohorts.

This study conducted in a real‐world sample of patients being treated for osteoporosis had a large sample size, up to 3 years of follow‐up, captured a sufficient number of cardiovascular and cerebrovascular events, and was replicated in two data systems. The cumulative risk of MI, stroke, composite cardiovascular disease outcome, and composite with death was similar across both denosumab and zoledronic acid treatment groups assessed by either RR or RD. These findings were consistent across subgroup analyses of patients with no CKD, competing risk analyses, and an ITT analysis. Accumulated evidence to date is not supportive of an increased risk of cardiovascular or cerebrovascular events among denosumab users.

## Author Contributions

**Leslie Spangler:** Conceptualization; formal analysis; methodology; writing – original draft; writing – review and editing. **Carrie M. Nielson:** Conceptualization; data curation; formal analysis; methodology; writing – review and editing. **M. Alan Brookhart:** Conceptualization; formal analysis; methodology; writing – review and editing. **Rohini K. Hernandez:** Conceptualization; data curation; formal analysis; methodology; visualization; writing – review and editing. **Robert Kees Stad:** Conceptualization; methodology; writing – review and editing. **Tzu‐Chieh Lin:** Conceptualization; formal analysis; methodology; writing – review and editing.

## Disclosures

MAB served on an Advisory Board for AbbVie, Amgen Inc, Atara Biotherapeutics, Brigham and Women's Hospital, Gilead, and Kite; and is a Major Stock stockholder for Target RWE. CMN and RKS were former employees and stockholder of Amgen Inc. LS, RKH, and TL are employees and stockholders of Amgen Inc.

## Supporting information


**Supplemental Table S1.** Study Enrollment Description by Database.
**Supplemental Table S2.** Disposition of Patients Who Initiated Denosumab or Zoledronic Acid Between 2010 and 2019, Followed Up for Up to 3 Years, With Each Censoring Reason.
**Supplemental Table S3.** Cumulative Risk by Treatment Group, Risk Ratio, and Risk Difference for Each Outcome.
**Supplemental Table S4.** Cumulative Risk by Treatment Group, Risk Ratio, and Risk Difference at 36 Months for Each Outcome, No Chronic Kidney Disease Subcohort.
**Supplemental Table S5.** Cumulative Risk by Treatment Group, Risk Ratio, and Risk Difference at 36 Months for Ischemic Stroke, MarketScan and Optum Cohorts.
**Supplemental Table S6.** Cumulative Risk by Treatment Group, Risk Ratio, and Risk Difference at 36 Months for Each Outcome, With Death Modeled as a Competing Risk, Optum Cohort.
**Supplemental Table S7.** Cumulative Risk by Treatment Group, Risk Ratio, and Risk Difference at 36 Months for Each Outcome, Optum Cohort, Intention‐to‐Treat Analysis.
**Supplemental Table S8.** Bounds on Corrected Estimates Under Assumptions of Unmeasured Confounder Strength for Each Observed Association at 36 Months, RR (95% CI).
**Supplemental Table S9.** Bounds on Corrected Estimates Under Assumptions of Unmeasured Confounder Strength for Each Observed Association at 36 Months, RR (95% CI).
**Supplemental Table S10.** Bounds on Corrected Estimates Under Assumptions of Unmeasured Confounder Strength for Each Observed Association at 36 Months, RR (95% CI).
**Supplemental Table S11.** Bounds on Corrected Estimates Under Assumptions of Unmeasured Confounder Strength for Each Observed Association at 36 Months, RR (95% CI).
**Supplemental Figure S1.** Cumulative risk plots with 95% confidence bands for each outcome.
**Supplemental Text S1.** Exposure definitions.

## Data Availability

Not applicable.
