# Supplementary material for: Cardiovascular Safety in Postmenopausal Women and Men With Osteoporosis Treated With Denosumab and Zoledronic Acid: A Post‐Authorization Safety Study
Source: JBMR Plus. 2023 Aug 21;7(10):e10793. doi: 10.1002/jbm4.10793 (PMC10556278; doi:10.1002/jbm4.10793)
Supplement: Supplementary file 1 — Supplemental Table S1. Study Enrollment Description by Database. Supplemental Table S2. Disposition of Patients Who Initiated Denosumab or Zoledronic Acid Between 2010 and 2019, Followed Up for Up to 3 Years, With Each Censoring Reason. Supplemental Table S3. Cumulative Risk by Treatment Group, Risk Ratio, and Risk Difference for Each Outcome. Supplemental Table S4. Cumulative Risk by Treatment Group, Risk Ratio, and Risk Difference at 36 Months for Each Outcome, No Chronic Kidney Disease Subcohort. Supplemental Table S5. Cumulative Risk by Treatment Group, Risk Ratio, and Risk Difference at 36 Months for Ischemic Stroke, MarketScan and Optum Cohorts. Supplemental Table S6. Cumulative Risk by Treatment Group, Risk Ratio, and Risk Difference at 36 Months for Each Outcome, With Death Modeled as a Competing Risk, Optum Cohort. Supplemental Table S7. Cumulative Risk by Treatment Group, Risk Ratio, and Risk Difference at 36 Months for Each Outcome, Optum Cohort, Intention‐to‐Treat Analysis. Supplemental Table S8. Bounds on Corrected Estimates Under Assumptions of Unmeasured Confounder Strength for Each Observed Association at 36 Months, RR (95% CI). Supplemental Table S9. Bounds on Corrected Estimates Under Assumptions of Unmeasured Confounder Strength for Each Observed Association at 36 Months, RR (95% CI). Supplemental Table S10. Bounds on Corrected Estimates Under Assumptions of Unmeasured Confounder Strength for Each Observed Association at 36 Months, RR (95% CI). Supplemental Table S11. Bounds on Corrected Estimates Under Assumptions of Unmeasured Confounder Strength for Each Observed Association at 36 Months, RR (95% CI). Supplemental Figure S1. Cumulative risk plots with 95% confidence bands for each outcome. Supplemental Text S1. Exposure definitions. [file JBM4-7-e10793-s001.docx]

**Supplementary Material**

**Cardiovascular Safety in Women and Men with Osteoporosis Treated with Denosumab and Zoledronic Acid: A Post-authorization Safety Study**

**Supplemental Table S1.** Study Enrollment Description by Database

| Criteria | Marketscan N (%) | Optum N (%) |
| --- | --- | --- |
| Total count of patients with age ≥ 55 during the study period | 58,794,048 | 27,271,675 |
|  |  |  |
| Inclusion criteriaa |  |  |
| Receipt of denosumab or zoledronic acid between October 1, 2010 and June 30, 2019 | 302,039 (100.0) | 260,776 (100.0) |
| Age ≥ 55 years at index date^b^ | 290,193 (96.1) | 258,014 (98.9) |
| At least 455 days of continuous enrollment preceding index date | 234,842 (77.8) | 199,130 (76.4) |
|  |  |  |
| Exclusion criteria^a^ |  |  |
| Diagnosis of Paget's disease of bone^c^ | 234,765 (77.7) | 198,930 (76.3) |
| Diagnosis of cancer (excluding non-melanoma skin cancer)^c^ | 161,009 (53.3) | 136,029 (52.2) |
| Treatment with chemotherapy^c^ | 140,322 (46.5) | 115,289 (44.2) |
| Treatment with hormonal therapy for cancer^c^ | 140,252 (46.4) | 115,255 (44.2) |
| Treatment with radiation or radiation therapy for cancer^c^ | 139,793 (46.3) | 114,924 (44.1) |
| History of stroke or MI^c^ | 133,772 (44.3) | 107,346 (41.2) |
| Previous administration for denosumab or zoledronic acid^d^ | 96,611 (32.0) | 73,127 (28.0) |

^a^Number remaining after the row criteria is applied. The denominator for percentage calculation is the total number of patients receiving denosumab or zoledronic acid.
^b^Index date is the date of first administration of denosumab or zoledronic acid.
^c^Excluded if the criterion was met during the 455 days preceding the index date.
^d^Excluded if the criterion was met any time before the index date.

MI = myocardial infarction.

**Supplemental Table S2.** Disposition of Patients Who Initiated Denosumab or Zoledronic Acid Between 2010 and 2019, Followed Up for Up to 3 Years, With Each Censoring Reason

| Subgroup | Median study follow-up^a^ (days) | Total censored before event^b^ n (%) | All-cause deaths n (%) | Medication discontinuation  n (%) | Medication switch n (%) | End of 3‑year follow-up period n (%) | End of available data in the database n (%) | End of enrollment in health plan n (%) | Cancer diagnosis or treatment n (%) | Paget’s disease n (%) |
| --- | --- | --- | --- | --- | --- | --- | --- | --- | --- | --- |
| **MarketScan** | | | | | | | | | | |
| **MI** | | | | | | | | | | |
| Overall  (N = 96,611) | 386 | 96,355 (99.7) | N/A | 38,630 (40.0) | 902 (0.9) | 5225 (5.4) | 8338 (8.6) | 44,457 (46.0) | 7192 (7.4) | 48 (0.0) |
| Denosumab (N = 56,068) | 260 | 55,921 (99.7) | N/A | 21,329 (38.0) | 145 (0.3) | 3317 (5.9) | 6259 (11.2) | 26,966 (48.1) | 4200 (7.5) | 4 (0.0) |
| Zoledronic acid (N = 40,543) | 426 | 40,434 (99.7) | N/A | 17,301 (42.7) | 757 (1.9) | 1908 (4.7) | 2079 (5.1) | 17,491 (43.1) | 2992 (7.4) | 44 (0.1) |
| **Stroke** | | | | | | | | | | |
| Overall  (N = 96,611) | 385 | 96,228 (99.6) | N/A | 38,589 (39.9) | 900 (0.9) | 5219 (5.4) | 8331 (8.6) | 44,382 (45.9) | 7188 (7.4) | 48 (0.0) |
| Denosumab (N = 56,068) | 260 | 55,836 (99.6) | N/A | 21,308 (38.0) | 145 (0.3) | 3317 (5.9) | 6257 (11.2) | 26,917 (48.0) | 4186 (7.5) | 3 (0.0) |
| Zoledronic acid (N = 40,543) | 426 | 40,392 (99.6) | N/A | 17,281 (42.6) | 755 (1.9) | 1902 (4.7) | 2074 (5.1) | 17,465 (43.1) | 3002 (7.4) | 45 (0.1) |
| **MI-stroke composite** | | | | | | | | | | |
| Overall  (N = 96,611) | 384 | 95,975 (99.3) | N/A | 38,457 (39.8) | 899 (0.9) | 5197 (5.4) | 8323 (8.6) | 44,310 (45.9) | 7163 (7.4) | 47 (0.0) |
| Denosumab (N = 56,068) | 259 | 55,690 (99.3) | N/A | 21,235 (37.9) | 145 (0.3) | 3300 (5.9) | 6251 (11.1) | 26,871 (47.9) | 4176 (7.4) | 3 (0.0) |
| Zoledronic acid (N = 40,543) | 426 | 40,285 (99.4) | N/A | 17,222 (42.5) | 754 (1.9) | 1897 (4.7) | 2072 (5.1) | 17,439 (43.0) | 2987 (7.4) | 44 (0.1) |
| **Optum** | | | | | | | | | | |
| **MI** | | | | | | | | | | |
| Overall  (N = 73,127) | 350 | 72,878 (99.7) | 1,162 (1.6) | 31,317 (42.8) | 594 (0.8) | 4143 (5.7) | 19,185 (26.2) | 11,235 (15.4) | 6317 (8.6) | 45 (0.1) |
| Denosumab (N = 49,551) | 248 | 49,401 (99.7) | 784 (1.6) | 21,007 (42.4) | 170 (0.3) | 2903 (5.9) | 14,300 (28.9) | 6842 (13.8) | 4121 (8.3) | 5 (0.0) |
| Zoledronic acid (N = 23,576) | 426 | 23,477 (99.6) | 378 (1.6) | 10,310 (43.7) | 424 (1.8) | 1240 (5.3) | 4885 (20.7) | 4393 (18.6) | 2196 (9.3) | 40 (0.2) |
| **Stroke** | | | | | | | | | | |
| Overall  (N = 73,127) | 350 | 72,795 (99.5) | 1150 (1.6) | 31,287 (42.8) | 594 (0.8) | 4138 (5.7) | 19,190 (26.2) | 11,212 (15.3) | 6287 (8.6) | 45 (0.1) |
| Denosumab (N = 49,551) | 247 | 49,322 (99.5) | 773 (1.6) | 20,977 (42.3) | 169 (0.3) | 2900 (5.9) | 14,302 (28.9) | 6819 (13.8) | 4099 (8.3) | 5 (0.0) |
| Zoledronic acid (N = 23,576) | 426 | 23,473 (99.6) | 377 (1.6) | 10,310 (43.7) | 425 (1.8) | 1238 (5.3) | 4888 (20.7) | 4393 (18.6) | 2188 (9.3) | 40 (0.2) |
| **MI-stroke composite** | | | | | | | | | | |
| Overall  (N = 73,127) | 349 | 72,548 (99.2) | 1115 (1.5) | 31,166 (42.6) | 593 (0.8) | 4119 (5.6) | 19,153 (26.2) | 11,165 (15.3) | 6270 (8.6) | 45 (0.1) |
| Denosumab (N = 49,551) | 247 | 49,174 (99.2) | 748 (1.5) | 20,910 (42.2) | 169 (0.3) | 2887 (5.8) | 14,277 (28.8) | 6789 (13.7) | 4089 (8.3) | 5 (0.0) |
| Zoledronic acid (N = 23,576) | 426 | 23,374 (99.1) | 367 (1.6) | 10,256 (43.5) | 424 (1.8) | 1232 (5.2) | 4876 (20.7) | 4376 (18.6) | 2181 (9.3) | 40 (0.2) |
| **Composite and all-cause mortality** | | | | | | | | | | |
| Overall  (N = 73,127) | 349 | 71,433 (97.7) | 0 | 31,164 (42.6) | 593 (0.8) | 4119 (5.6) | 19,131 (26.2) | 10,257 (14.0) | 6270 (8.6) | 45 (0.1) |
| Denosumab (N = 49,551) | 247 | 48,426 (97.7) | 0 | 20,909 (42.2) | 169 (0.3) | 2887 (5.8) | 14,260 (28.8) | 6199 (12.5) | 4089 (8.3) | 5 (0.0) |
| Zoledronic acid (N = 23,576) | 426 | 23,007 (97.6) | 0 | 10,255 (43.5) | 424 (1.8) | 1232 (5.2) | 4871 (20.7) | 4058 (17.2) | 2181 (9.3) | 40 (0.2) |

^a^Follow-up days are inclusive of the outcome day or censored day, whichever occurred earlier.

^b^The total censored events may not be equal to the sum of each reason as more than one censored event may have occurred on the same day.

MI = myocardial infarction; N/A = not applicable.

**Supplemental Table S3.** Cumulative Risk by Treatment Group, Risk Ratio, and Risk Difference for Each Outcome

|  | | | **6 months** | | | | **12 months** | | | **36 months** | | |
| --- | --- | --- | --- | --- | --- | --- | --- | --- | --- | --- | --- | --- |
| Outcome | Treatment | CR per 100 (95% CI) | | RR  (95% CI) | RD per 100  95% CI) | CR per 100 (95% CI) | | RR  (95% CI) | RD per 100 (95% CI) | CR per 100 (95% CI) | RR  (95% CI) | RD per 100 (95% CI) |
| **MarketScan** |  |  | |  |  |  | |  |  |  |  |  |
| MI | Zoledronic acid | 0.16  (0.11, 0.21) | |  |  | 0.30  (0.22, 0.37) | |  |  | 0.51  (0.37, 0.66) |  |  |
| MI | Denosumab | 0.11  (0.08, 0.14) | | 0.69  (0.41, 0.97) | -0.05  (-0.11, 0.01) | 0.23  (0.18, 0.28) | | 0.78  (0.51, 1.05) | -0.06  (-0.16, 0.03) | 0.63  (0.48, 0.77) | 1.22  (0.77, 1.66) | 0.11  (-0.09, 0.32) |
| Stroke | Zoledronic acid | 0.20  (0.13, 0.27) | |  |  | 0.36  (0.28, 0.45) | |  |  | 1.14  (0.76, 1.52) |  |  |
| Stroke | Denosumab | 0.19  (0.15, 0.23) | | 0.95  (0.58, 1.32) | -0.01  (-0.09, 0.07) | 0.32  (0.26, 0.38) | | 0.89  (0.63, 1.14) | -0.04  (-0.14, 0.06) | 1.14  (0.90, 1.38) | 1.00  (0.61, 1.40) | 0.00  (-0.45, 0.45) |
| MI-stroke composite | Zoledronic acid | 0.36  (0.27, 0.44) | |  |  | 0.66  (0.55, 0.77) | |  |  | 1.75  (1.31, 2.20) |  |  |
| MI-stroke composite | Denosumab | 0.30  (0.25, 0.35) | | 0.85  (0.61, 1.09) | -0.05  (-0.15, 0.04) | 0.55  (0.48, 0.63) | | 0.84  (0.65, 1.03) | -0.11  (-0.24, 0.03) | 1.79  (1.51, 2.08) | 1.02  (0.71, 1.33) | 0.04  (-0.49, 0.57) |
| **Optum** |  |  | |  |  |  | |  |  |  |  |  |
| MI | Zoledronic acid | 0.18  (0.12, 0.24) | |  |  | 0.42  (0.32, 0.52) | |  |  | 1.01  (0.73, 1.29) |  |  |
| MI | Denosumab | 0.15  (0.12, 0.19) | | 0.85  (0.49, 1.21) | -0.03  (-0.10, 0.05) | 0.28  (0.22, 0.33) | | 0.66  (0.45, 0.87) | -0.14  (-0.26, -0.03) | 0.98  (0.77, 1.20) | 0.97  (0.63, 1.32) | -0.03  (-0.37, 0.32) |
| Stroke | Zoledronic acid | 0.17  (0.11, 0.23) | |  |  | 0.37  (0.28, 0.46) | |  |  | 1.39  (0.94, 1.85) |  |  |
| Stroke | Denosumab | 0.23  (0.18, 0.28) | | 1.38  (0.82, 1.94) | 0.06  (-0.01, 0.14) | 0.44  (0.37, 0.51) | | 1.18  (0.84, 1.53) | 0.07  (-0.05, 0.18) | 1.21  (1.05, 1.37) | 0.87  (0.56, 1.17) | -0.19  (-0.67, 0.30) |
| MI-stroke composite | Zoledronic acid | 0.35  (0.26, 0.43) | |  |  | 0.79  (0.65, 0.92) | |  |  | 2.39  (1.85, 2.93) |  |  |
| MI-stroke composite | Denosumab | 0.38  (0.33, 0.44) | | 1.10  (0.78, 1.43) | 0.04  (-0.07, 0.14) | 0.71  (0.62, 0.80) | | 0.90  (0.71, 1.10) | -0.08  (-0.24, 0.09) | 2.18  (1.90, 2.45) | 0.91  (0.68, 1.14) | -0.21  (-0.82, 0.39) |
| MI-stroke-death | Zoledronic acid | 0.99  (0.83, 1.14) | |  |  | 2.35  (2.10, 2.60) | |  |  | 8.01  (6.92, 9.10) |  |  |
| MI-stroke-death | Denosumab | 1.02  (0.93, 1.12) | | 1.03  (0.84, 1.22) | 0.03  (-0.15, 0.21) | 2.07  (1.91, 2.23) | | 0.88  (0.76, 1.00) | -0.28  (-0.58, 0.02) | 7.41  (6.86, 7.96) | 0.93  (0.78, 1.07) | -0.60  (-1.82, 0.62) |

All results are from inverse probability of treatment and censoring weighted estimation functions. Propensity score trimming by 1% was applied. CR is per 100 individuals, and RD is the difference between CR values. Zoledronic acid treatment group was considered as the reference group.

CR = cumulative risk; MI = myocardial infarction; RD = risk difference; RR = risk ratio.

**Supplemental Table S4.** Cumulative Risk by Treatment Group, Risk Ratio, and Risk Difference at 36 Months for Each Outcome, No Chronic Kidney Disease Subcohort

| Outcome | Treatment | CR per 100 (95% CI) | RR (95% CI) | RD per 100 (95% CI) |
| --- | --- | --- | --- | --- |
| **MarketScan** |  |  |  |  |
| MI | Zoledronic acid | 0.49 (0.34, 0.65) |  |  |
| MI | Denosumab | 0.57 (0.42, 0.72) | 1.16 (0.68, 1.64) | 0.08 (-0.14, 0.30) |
| Stroke | Zoledronic acid | 1.10 (0.68, 1.52) |  |  |
| Stroke | Denosumab | 0.98 (0.77, 1.20) | 0.89 (0.50, 1.28) | -0.12 (-0.59, 0.35) |
| MI-stroke composite | Zoledronic acid | 1.59 (1.12, 2.06) |  |  |
| MI-stroke composite | Denosumab | 1.55 (1.28, 1.81) | 0.97 (0.64, 1.31) | -0.04 (-0.58, 0.50) |
| **Optum** |  |  |  |  |
| MI | Zoledronic acid | 0.95 (0.66, 1.24) |  |  |
| MI | Denosumab | 0.80 (0.62, 0.98) | 0.84 (0.52, 1.17) | -0.15 (-0.49, 0.20) |
| Stroke | Zoledronic acid | 1.30 (0.80, 1.79) |  |  |
| Stroke | Denosumab | 1.08 (0.91, 1.25) | 0.83 (0.49, 1.18) | -0.22 (-0.74, 0.31) |
| MI-stroke composite | Zoledronic acid | 2.23 (1.66, 2.81) |  |  |
| MI-stroke composite | Denosumab | 1.87 (1.62, 2.12) | 0.84 (0.59, 1.08) | -0.36 (-0.99, 0.27) |
| MI-stroke-death | Zoledronic acid | 6.90 (5.78, 8.02) |  |  |
| MI-stroke-death | Denosumab | 6.05 (5.51, 6.59) | 0.88 (0.71, 1.04) | -0.84 (-2.09, 0.40) |

All results are from inverse probability of treatment and censoring weighted estimation functions. Propensity score trimming by 1% was applied. CR is per 100 individuals, and RD is the difference between CR values.

CR = cumulative risk; MI = myocardial infarction; RD = risk difference; RR = risk ratio.

**Supplemental Table S5.** Cumulative Risk by Treatment Group, Risk Ratio, and Risk Difference at 36 Months for Ischemic Stroke, MarketScan and Optum Cohorts

| Treatment | CR per 100 (95% CI) | RR  (95% CI) | RD per 100 (95% CI) |
| --- | --- | --- | --- |
| **MarketScan** | | | |
| Zoledronic acid | 1.00 (0.63, 1.36) |  |  |
| Denosumab | 1.00 (0.77, 1.23) | 1.00 (0.57, 1.44) | 0.00 (-0.43, 0.43) |
| **Optum** | | | |
| Zoledronic acid | 1.30 (0.85, 1.76) |  |  |
| Denosumab | 1.04 (0.89, 1.20) | 0.80 (0.50, 1.10) | -0.26 (-0.74, 0.22) |

All results are from inverse probability of treatment and censoring weighted estimation functions. Propensity score trimming by 1% was applied. CR is per 100 individuals, and RD is the difference between CR values. Reference group: zoledronic acid treatment.

CR = cumulative risk; RD = risk difference; RR = risk ratio.

**Supplemental Table S6.** Cumulative Risk by Treatment Group, Risk Ratio, and Risk Difference at 36 Months for Each Outcome, With Death Modeled as a Competing Risk, Optum Cohort

| Outcome | Treatment | CR per 100  (95% CI) | RR   (95% CI) | RD per 100  (95% CI) |
| --- | --- | --- | --- | --- |
| MI | Zoledronic acid | 0.97 (0.70, 1.24) |  |  |
| MI | Denosumab | 1.02 (0.83, 1.21) | 1.06 (0.70, 1.41) | 0.06 (-0.27, 0.38) |
| Stroke | Zoledronic acid | 1.29 (0.87, 1.70) |  |  |
| Stroke | Denosumab | 1.33 (1.17, 1.49) | 1.03 (0.68, 1.39) | 0.04 (-0.40, 0.49) |
| MI-stroke composite | Zoledronic acid | 2.25 (1.75, 2.75) |  |  |
| MI-stroke composite | Denosumab | 2.32 (2.06, 2.58) | 1.03 (0.78, 1.29) | 0.07 (-0.49, 0.64) |

All results are from inverse probability of treatment and censoring weighted estimation functions. Propensity score trimming by 1% was applied. CR is per 100 individuals, and RD is the difference between CR values.

CR = cumulative risk; RD = risk difference; RR = risk ratio; MI = myocardial infarction.

**Supplemental Table S7.** Cumulative Risk by Treatment Group, Risk Ratio, and Risk Difference at 36 Months for Each Outcome, Optum Cohort, Intention-to-Treat Analysis

| Outcome | Treatment | CR per 100  (95% CI) | | | RR   (95% CI) | | RD per 100  (95% CI) |
| --- | --- | --- | --- | --- | --- | --- | --- |
| **Optum** |  |  | | |  | |  |
| MI | Zoledronic acid | 1.26 (1.08, 1.44) | | |  | |  |
| MI | Denosumab | 1.18 (1.04, 1.33) | | | 0.94 (0.76, 1.11) | | -0.08 (-0.31, 0.15) |
| Stroke | Zoledronic acid | 1.75 (1.53, 1.97) | | |  | |  |
| Stroke | Denosumab | 1.58 (1.45, 1.71) | | | 0.90 (0.77, 1.04) | | -0.17 (-0.43, 0.09) |
| MI-stroke composite | Zoledronic acid | 2.98 (2.70, 3.26) | | |  | |  |
| MI-stroke composite | Denosumab | 2.73 (2.54, 2.92) | | | 0.92 (0.81, 1.02) | | -0.25 (-0.59, 0.09) |
| MI-stroke-death | Zoledronic acid | 9.65 (9.12, 10.18) | | |  | |  |
| MI-stroke-death | Denosumab | 9.96 (9.62, 10.30) | | | 1.03 (0.97, 1.10) | | 0.31 (-0.32, 0.94) |
| **MarketScan** |  | |  |  | |  | |
| MI | Zoledronic acid | | 0.94 (0.77, 1.11) |  | |  | |
| MI | Denosumab | | 0.89 (0.76, 1.01) | 0.94 (0.72, 1.16) | | -0.06 (-0.27, 0.16) | |
| Stroke | Zoledronic acid | | 1.24 (1.04, 1.44) |  | |  | |
| Stroke | Denosumab | | 1.21 (1.06, 1.36) | 0.98 (0.78, 1.17) | | -0.03 (-0.28, 0.22) | |
| MI-stroke composite | Zoledronic acid | | 2.17 (1.90, 2.43) |  | |  | |
| MI-stroke composite | Denosumab | | 2.05 (1.86, 2.25) | 0.95 (0.80, 1.09) | | -0.11 (-0.44, 0.21) | |

All results are from inverse probability of treatment and censoring weighted estimation functions. Propensity score trimming by 1% was applied. CR is per 100 individuals, and RD is the difference between CR values.
CR = cumulative risk; RD = risk difference; RR = risk ratio; MI = myocardial infarction.

**Tables S8-S11 – Quantitative Bias Analysis**

**Supplemental Table S8.** Bounds on Corrected Estimates Under Assumptions of Unmeasured Confounder Strength for Each Observed Association at 36 Months, RR (95% CI)

|  | RR Treatment | | | | | | | | |
| --- | --- | --- | --- | --- | --- | --- | --- | --- | --- |
| RR Disease | 1.3 | 1.5 | 1.8 | 2 | 2.5 | 3 | 3.5 | 4 | 5 |
| **MI, MarketScan, Observed RR =** **1.22 (95% CI: 0.77, 1.66)** | | | | | | | | | |
| 1.3 | 1.16  (0.73, 1.57) | 1.13  (0.71, 1.53) | 1.09  (0.69, 1.49) | 1.08  (0.68, 1.47) | 1.05  (0.66, 1.43) | 1.03  (0.65, 1.40) | 1.02  (0.64, 1.39) | 1.01  (0.64, 1.37) | 0.99  (0.63, 1.35) |
| 1.5 | 1.13  (0.71, 1.53) | 1.08  (0.68, 1.48) | 1.04  (0.66, 1.41) | 1.02  (0.64, 1.38) | 0.98  (0.62, 1.33) | 0.95  (0.60, 1.29) | 0.93  (0.59, 1.26) | 0.92  (0.58, 1.25) | 0.89  (0.56, 1.22) |
| 1.8 | 1.09  (0.69, 1.49) | 1.04  (0.66, 1.41) | 0.98  (0.62, 1.33) | 0.95  (0.60, 1.29) | 0.89  (0.56, 1.22) | 0.86  (0.54, 1.17) | 0.83  (0.53, 1.13) | 0.81  (0.51, 1.11) | 0.79  (0.50, 1.07) |
| 2 | 1.08  (0.68, 1.47) | 1.02  (0.64, 1.38) | 0.95  (0.60, 1.29) | 0.92  (0.58, 1.25) | 0.85  (0.54, 1.16) | 0.81  (0.51, 1.11) | 0.78  (0.50, 1.07) | 0.76  (0.48, 1.04) | 0.73  (0.46, 1.00) |
| 2.5 | 1.05  (0.66, 1.43) | 0.98  (0.62, 1.33) | 0.89  (0.56, 1.22) | 0.85  (0.54, 1.16) | 0.78  (0.49, 1.06) | 0.73  (0.46, 1.00) | 0.70  (0.44, 0.95) | 0.67  (0.42, 0.91) | 0.63  (0.40, 0.86) |
| 3 | 1.03  (0.65, 1.40) | 0.95  (0.60, 1.29) | 0.86  (0.54, 1.17) | 0.81  (0.51, 1.11) | 0.73  (0.46, 1.00) | 0.68  (0.43, 0.92) | 0.64  (0.40, 0.87) | 0.61  (0.38, 0.83) | 0.57  (0.36, 0.77) |
| 3.5 | 1.02  (0.64, 1.39) | 0.93  (0.59, 1.26) | 0.83  (0.53, 1.13) | 0.78  (0.50, 1.07) | 0.70  (0.44, 0.95) | 0.64  (0.40, 0.87) | 0.60  (0.38, 0.81) | 0.57  (0.36, 0.77) | 0.52  (0.33, 0.71) |
| 4 | 1.01  (0.64, 1.37) | 0.92  (0.58, 1.25) | 0.81  (0.51, 1.11) | 0.76  (0.48, 1.04) | 0.67  (0.42, 0.91) | 0.61  (0.38, 0.83) | 0.57  (0.36, 0.77) | 0.53 (0.34, 0.73) | 0.49  (0.31, 0.66) |
| 5 | 0.99  (0.63, 1.35) | 0.89  (0.56, 1.22) | 0.79  (0.50, 1.07) | 0.73  (0.46, 1.00) | 0.63  (0.40, 0.86) | 0.57  (0.36, 0.77) | 0.52  (0.33, 0.71) | 0.49  (0.31, 0.66) | 0.44  (0.28, 0.60) |

MI = myocardial infarction; RR = risk ratio.

RR disease: strength of association between an unmeasured confounder and the outcome.
RR treatment: strength of association between an unmeasured confounder and the treatment

**Supplemental Table S9.** Bounds on Corrected Estimates Under Assumptions of Unmeasured Confounder Strength for Each Observed Association at 36 Months, RR (95% CI)

|  | RR Treatment | | | | | | | | |
| --- | --- | --- | --- | --- | --- | --- | --- | --- | --- |
| RR Disease | 1.3 | 1.5 | 1.8 | 2 | 2.5 | 3 | 3.5 | 4 | 5 |
| **MI, Optum, Observed RR = 0.97 (95% CI: 0.63, 1.32)** | | | | | | | | | |
| 1.3 | 0.92  (0.60, 1.25) | 0.90  (0.58, 1.22) | 0.87  (0.57, 1.18) | 0.86  (0.56, 1.17) | 0.84  (0.54, 1.14) | 0.82  (0.53, 1.12) | 0.81  (0.53, 1.10) | 0.80  (0.52, 1.09) | 0.79  (0.51, 1.08) |
| 1.5 | 0.90  (0.58, 1.22) | 0.86  (0.56, 1.17) | 0.83  (0.54, 1.12) | 0.81  (0.52, 1.10) | 0.78  (0.50, 1.06) | 0.75  (0.49, 1.03) | 0.74  (0.48, 1.01) | 0.73  (0.47, 0.99) | 0.71  (0.46, 0.97) |
| 1.8 | 0.87  (0.57, 1.18) | 0.83  (0.54, 1.12) | 0.78  (0.51, 1.06) | 0.75  (0.49, 1.03) | 0.71  (0.46, 0.97) | 0.68  (0.44, 0.93) | 0.66  (0.43, 0.90) | 0.65  (0.42, 0.88) | 0.63  (0.41, 0.85) |
| 2 | 0.86  (0.56, 1.17) | 0.81  (0.52, 1.10) | 0.75  (0.49, 1.03) | 0.73  (0.47, 0.99) | 0.68  (0.44, 0.92) | 0.65  (0.42, 0.88) | 0.62  (0.40, 0.85) | 0.61  (0.39, 0.82) | 0.58  (0.38, 0.79) |
| 2.5 | 0.84  (0.54, 1.14) | 0.78  (0.50, 1.06) | 0.71  (0.46, 0.97) | 0.68  (0.44, 0.92) | 0.62  (0.40, 0.84) | 0.58  (0.38, 0.79) | 0.55  (0.36, 0.75) | 0.53  (0.35, 0.73) | 0.50  (0.33, 0.69) |
| 3 | 0.82  (0.53, 1.12) | 0.75  (0.49, 1.03) | 0.68  (0.44, 0.93) | 0.65  (0.42, 0.88) | 0.58  (0.38, 0.79) | 0.54  (0.35, 0.73) | 0.51  (0.33, 0.69) | 0.48  (0.32, 0.66) | 0.45  (0.29, 0.62) |
| 3.5 | 0.81  (0.53, 1.10) | 0.74  (0.48, 1.01) | 0.66  (0.43, 0.90) | 0.62  (0.40, 0.85) | 0.55  (0.36, 0.75) | 0.51  (0.33, 0.69) | 0.48  (0.31, 0.65) | 0.45  (0.29, 0.61) | 0.42  (0.27, 0.57) |
| 4 | 0.80  (0.52, 1.09) | 0.73  (0.47, 0.99) | 0.65  (0.42, 0.88) | 0.61  (0.39, 0.82) | 0.53  (0.35, 0.73) | 0.48  (0.32, 0.66) | 0.45  (0.29, 0.61) | 0.42  (0.28, 0.58) | 0.39  (0.25, 0.53) |
| 5 | 0.79  (0.51, 1.08) | 0.71  (0.46, 0.97) | 0.63  (0.41, 0.85) | 0.58  (0.38, 0.79) | 0.50  (0.33, 0.69) | 0.45  (0.29, 0.62) | 0.42  (0.27, 0.57) | 0.39  (0.25, 0.53) | 0.35  (0.23, 0.48) |

MI = myocardial infarction; RR = risk ratio.

RR disease: strength of association between an unmeasured confounder and the outcome.
RR treatment: strength of association between an unmeasured confounder and the treatment

**Supplemental Table S10.** Bounds on Corrected Estimates Under Assumptions of Unmeasured Confounder Strength for Each Observed Association at 36 Months, RR (95% CI)

|  | RR Treatment | | | | | | | | |
| --- | --- | --- | --- | --- | --- | --- | --- | --- | --- |
| RR Disease | 1.3 | 1.5 | 1.8 | 2 | 2.5 | 3 | 3.5 | 4 | 5 |
| **Stroke, MarketScan, Observed RR = 1.00 (95% CI: 0.61, 1.40)** | | | | | | | | | |
| 1.3 | 0.95  (0.58, 1.33) | 0.92  (0.56, 1.29) | 0.90  (0.55, 1.26) | 0.88  (0.54, 1.24) | 0.86  (0.53, 1.21) | 0.85  (0.52, 1.18) | 0.84  (0.51, 1.17) | 0.83  (0.50, 1.16) | 0.82  (0.50, 1.14) |
| 1.5 | 0.92  (0.56, 1.29) | 0.89  (0.54, 1.24) | 0.85  (0.52, 1.19) | 0.83  (0.51, 1.17) | 0.80  (0.49, 1.12) | 0.78  (0.47, 1.09) | 0.76  (0.46, 1.07) | 0.75  (0.46, 1.05) | 0.73  (0.45, 1.03) |
| 1.8 | 0.90  (0.55, 1.26) | 0.85  (0.52, 1.19) | 0.80  (0.49, 1.12) | 0.78  (0.47, 1.09) | 0.73  (0.45, 1.03) | 0.70  (0.43, 0.99) | 0.68  (0.42, 0.96) | 0.67  (0.41, 0.93) | 0.64  (0.39, 0.90) |
| 2 | 0.88  (0.54, 1.24) | 0.83  (0.51, 1.17) | 0.78  (0.47, 1.09) | 0.75  (0.46, 1.05) | 0.70  (0.43, 0.98) | 0.67  (0.41, 0.93) | 0.64  (0.39, 0.90) | 0.62  (0.38, 0.87) | 0.60  (0.37, 0.84) |
| 2.5 | 0.86  (0.53, 1.21) | 0.80  (0.49, 1.12) | 0.73  (0.45, 1.03) | 0.70  (0.43, 0.98) | 0.64  (0.39, 0.90) | 0.60  (0.37, 0.84) | 0.57  (0.35, 0.80) | 0.55  (0.34, 0.77) | 0.52  (0.32, 0.73) |
| 3 | 0.85  (0.52, 1.18) | 0.78  (0.47, 1.09) | 0.70  (0.43, 0.99) | 0.67  (0.41, 0.93) | 0.60  (0.37, 0.84) | 0.56  (0.34, 0.78) | 0.52  (0.32, 0.73) | 0.50  (0.30, 0.70) | 0.47  (0.28, 0.65) |
| 3.5 | 0.84  (0.51, 1.17) | 0.76  (0.46, 1.07) | 0.68  (0.42, 0.96) | 0.64  (0.39, 0.90) | 0.57  (0.35, 0.80) | 0.52  (0.32, 0.73) | 0.49  (0.30, 0.69) | 0.46  (0.28, 0.65) | 0.43  (0.26, 0.60) |
| 4 | 0.83  (0.50, 1.16) | 0.75  (0.46, 1.05) | 0.67  (0.41, 0.93) | 0.62  (0.38, 0.87) | 0.55  (0.34, 0.77) | 0.50  (0.30, 0.70) | 0.46  (0.28, 0.65) | 0.44  (0.27, 0.61) | 0.40  (0.24, 0.56) |
| 5 | 0.82  (0.50, 1.14) | 0.73  (0.45, 1.03) | 0.64  (0.39, 0.90) | 0.60  (0.37, 0.84) | 0.52  (0.32, 0.73) | 0.47  (0.28, 0.65) | 0.43  (0.26, 0.60) | 0.40  (0.24, 0.56) | 0.36  (0.22, 0.50) |

RR = risk ratio.

RR disease: strength of association between an unmeasured confounder and the outcome.
RR treatment: strength of association between an unmeasured confounder and the treatment

**Supplemental Table S11.** Bounds on Corrected Estimates Under Assumptions of Unmeasured Confounder Strength for Each Observed Association at 36 Months, RR (95% CI)

|  | RR Treatment | | | | | | | | |
| --- | --- | --- | --- | --- | --- | --- | --- | --- | --- |
| RR Disease | 1.3 | 1.5 | 1.8 | 2 | 2.5 | 3 | 3.5 | 4 | 5 |
| **Stroke, Optum, Observed RR = 0.87 (95% CI: 0.56, 1.17)** | | | | | | | | | |
| 1.3 | 0.82  (0.53, 1.11) | 0.80  (0.52, 1.08) | 0.78  (0.50, 1.05) | 0.77  (0.50, 1.03) | 0.75  (0.48, 1.01) | 0.74  (0.47, 0.99) | 0.73  (0.47, 0.98) | 0.72  (0.46, 0.97) | 0.71  (0.46, 0.95) |
| 1.5 | 0.80  (0.52, 1.08) | 0.77  (0.50, 1.04) | 0.74  (0.48, 1.00) | 0.72  (0.47, 0.98) | 0.70  (0.45, 0.94) | 0.68  (0.44, 0.91) | 0.66  (0.43, 0.89) | 0.65  (0.42, 0.88) | 0.64  (0.41, 0.86) |
| 1.8 | 0.78  (0.50, 1.05) | 0.74  (0.48, 1.00) | 0.70  (0.45, 0.94) | 0.68  (0.44, 0.91) | 0.64  (0.41, 0.86) | 0.61  (0.39, 0.82) | 0.59  (0.38, 0.80) | 0.58  (0.37, 0.78) | 0.56  (0.36, 0.75) |
| 2 | 0.77  (0.50, 1.03) | 0.72  (0.47, 0.98) | 0.68  (0.44, 0.91) | 0.65  (0.42, 0.88) | 0.61  (0.39, 0.82) | 0.58  (0.37, 0.78) | 0.56  (0.36, 0.75) | 0.54  (0.35, 0.73) | 0.52  (0.34, 0.70) |
| 2.5 | 0.75  (0.48, 1.01) | 0.70  (0.45, 0.94) | 0.64  (0.41, 0.86) | 0.61  (0.39, 0.82) | 0.56  (0.36, 0.75) | 0.52  (0.34, 0.70) | 0.50  (0.32, 0.67) | 0.48  (0.31, 0.64) | 0.45  (0.29, 0.61) |
| 3 | 0.74  (0.47, 0.99) | 0.68  (0.44, 0.91) | 0.61  (0.39, 0.82) | 0.58  (0.37, 0.78) | 0.52  (0.34, 0.70) | 0.48  (0.31, 0.65) | 0.46  (0.29, 0.61) | 0.44  (0.28, 0.58) | 0.41  (0.26, 0.55) |
| 3.5 | 0.73  (0.47, 0.98) | 0.66  (0.43, 0.89) | 0.59  (0.38, 0.80) | 0.56  (0.36, 0.75) | 0.50  (0.32, 0.67) | 0.46  (0.29, 0.61) | 0.43  (0.27, 0.57) | 0.40  (0.26, 0.54) | 0.37  (0.24, 0.50) |
| 4 | 0.72  (0.46, 0.97) | 0.65  (0.42, 0.88) | 0.58  (0.37, 0.78) | 0.54  (0.35, 0.73) | 0.48  (0.31, 0.64) | 0.44  (0.28, 0.58) | 0.40  (0.26, 0.54) | 0.38  (0.25, 0.51) | 0.35  (0.22, 0.47) |
| 5 | 0.71  (0.46, 0.95) | 0.64  (0.41, 0.86) | 0.56  (0.36, 0.75) | 0.52  (0.34, 0.70) | 0.45  (0.29, 0.61) | 0.41  (0.26, 0.55) | 0.37  (0.24, 0.50) | 0.35  (0.22, 0.47) | 0.31  (0.20, 0.42) |

RR = risk ratio.

RR disease: strength of association between an unmeasured confounder and the outcome.
RR treatment: strength of association between an unmeasured confounder and the treatment.

**Supplemental Figure S1.** Cumulative risk plots with 95% confidence bands for each outcome.


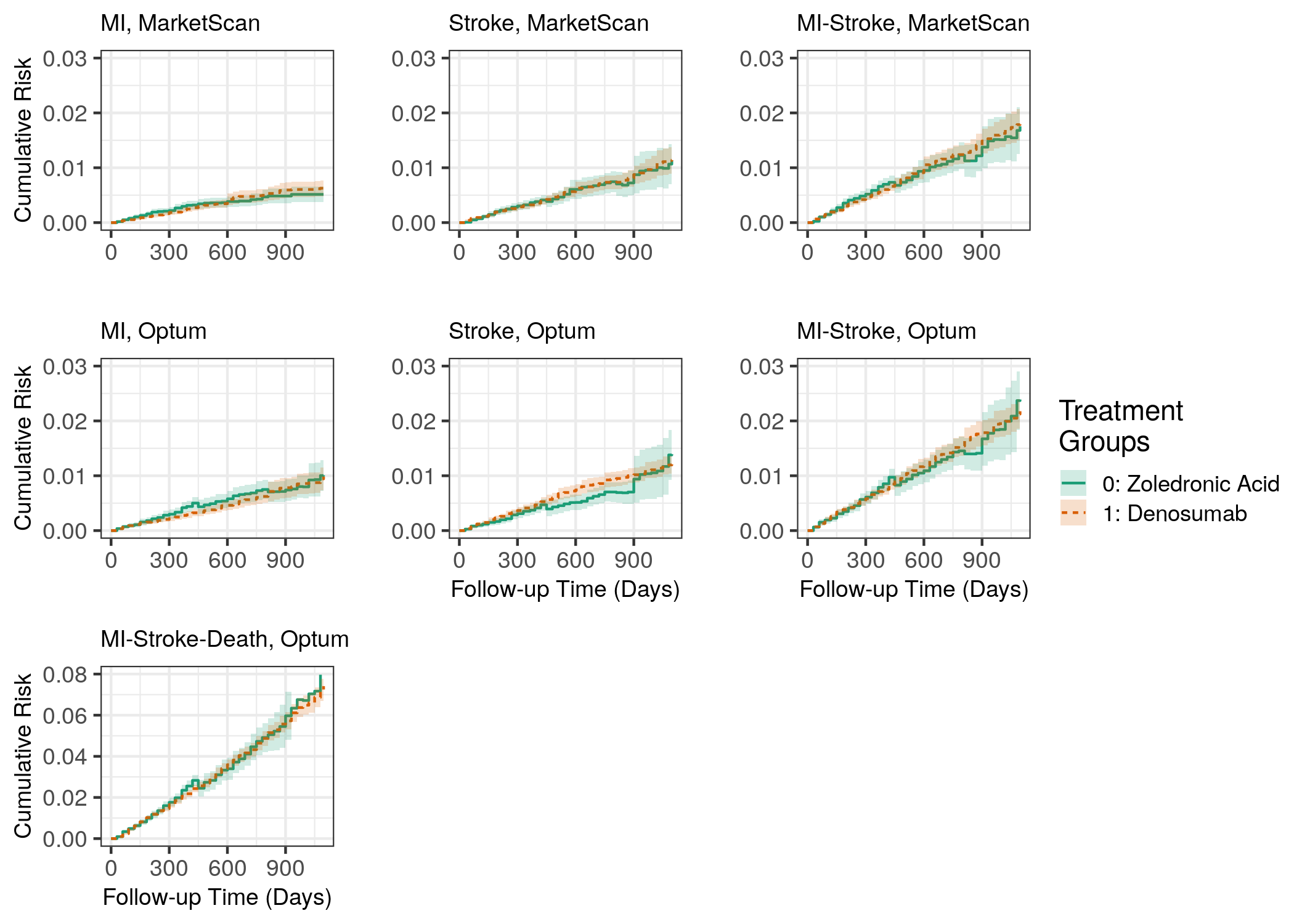


MI = myocardial infarction

# Supplemental Text S1. Exposure definitions

The dosing interval for denosumab 60 mg is once every 6 months (182 days). Patients were considered continuously exposed to denosumab from the date of first administration through the end of an allowable gap period of 60 days after the standard dosing interval (182 days) after the last recorded denosumab administration. Denosumab was identified using Healthcare Common Procedure Coding System

(HCPCS) codes:

- C9272 - Injection, denosumab, 1 mg, effective October 1, 2010, to December 31, 2011;
- J0897 - Injection, denosumab, 1 mg, effective January 1, 2012; and
- National Drug Code (NDC): 55513071001 – strength 60 mg.

The osteoporosis dosing interval for zoledronic acid is 5 mg once a year (365 days). Patients were considered continuously exposed to zoledronic acid from the date of first administration through the end of an allowable gap period of 60 days after the standard dosing interval (365 days) after the last recorded zoledronic acid administration. Zoledronic acid was identified using current HCPCS codes:

- J3488 - Injection, zoledronic acid (Reclast) 1 mg effective from January 1, 2008;
- J3489 – Injection, zoledronic acid, 1 mg effective from January 1, 2014;
- Q2051 - Injection, zoledronic acid, not otherwise specified, 1 mg effective from July 1, 2013;
- Q4095 - Injection, zoledronic acid (Reclast) 1 mg effective from July 1, 2007; and
- Current NDCs: 00078043561, 23155018631, 25021083082, 35356035101, 42023016301, 43598033111, 55111068852, 00409422801, 51991006498, 63323096600, 67457061910, 67457079410 – strength 5 mg.

The HCPCS codes specific for denosumab (C9272 and J0897) and the later codes for zoledronic acid (Q2051 and J3488) are not specific to osteoporosis (Prolia/Reclast) vs cancer (XGEVA/Zometa) treatment. The use of these codes was acceptable because patients with cancer at baseline (defined as 455 days before the index date [excluding index date]) were excluded and the study included censoring during follow-up.
